# Operators’ Ease and Satisfaction in Restoring Class II Cavities With Sectional Matrix Versus Circumferential Matrix System at Qassim University Dental Clinics

**DOI:** 10.7759/cureus.20957

**Published:** 2022-01-05

**Authors:** Mohammed Almushayti, Bilal Arjumand

**Affiliations:** 1 College of Dentistry, Qassim University, Buraydah, SAU; 2 Department of Conservative Dentistry, College of Dentistry, Qassim University, Buraydah, SAU

**Keywords:** sectional matrix band system, circumferential matrix band system, resin composite, proximal contact, class ii, operator satisfaction, matrix band system

## Abstract

Objectives: This study aimed to investigate the operator’s ease, satisfaction, and comfort of using a circumferential matrix system and sectional matrix system on the proximal contact points and contours when restoring class II cavities in posterior teeth.

Materials and methods: This cross-sectional study was performed at the clinics in the Department of Conservative Dental Sciences, College of Dentistry, Qassim University. A total of 105 dental students randomly participated in this study to restore a class II cavity with direct composite resin restoration. Operators' comfort and satisfaction were evaluated according to their assessment of the contact points they reproduced and the emergence profiles of restorations, using a circumferential matrix system and sectional matrix system.

Results: Out of 105 operators, 57 were satisfied with using circumferential matrix bands for most of their cases while 78 of the operators were satisfied with sectional matrix bands. There were no significant differences between operator satisfaction and the use of circumferential matrix band system (P > 0.05) and sectional matrix band system (P = 0.134) but there was a significant difference between difficulty levels in the use of both matrix band systems (P < 0.05; P = 0.000).

Conclusion: Circumferential and sectional matrix band systems showed no significant differences with operators' satisfaction during restoring class II cavities in posterior teeth but using a sectional matrix band system was considered easier than using a circumferential matrix band system.

## Introduction

A matrix band system is a properly contoured piece of metal or other material that helps to support and give form to the restoration during its placement and hardening [1]. Making optimum contact points with direct restoration still remains a difficult target to achieve [2,3]. A contact that is not adequate usually results in food impaction, periodontal disease, and movement of the tooth [4,5]. Matrix band system is used to restore cavities with missing proximal walls [3].

Matrices, historically, have been categorized as circumferential or sectional and were used with a holder or retainer. Dr. Joseph B.F. Tofflemire in 1946 introduced the Tofflemire retainer and band (also known as the universal matrix system) and is still in use today. It produces good contours and contacts for use with amalgam and can also be employed for insertion of composite resin, but more recently developed matrix systems like sectional matrix band (SMB) systems have been proven more clinically efficacious, especially for the attainment of inter-proximal contacts [6,7].

The primary function of a matrix band is to compensate for missing walls and thus provide containment of the filling material [7]. Matrix band systems have evolved over time and newer systems are made to give ease of use to operators and also cover for flaws and difficulty in older systems.

Circumferential matrix band (CMB) system has been conventionally used to restore class II cavities [5]. SMB system is relatively new and has been shown to produce anatomically optimum contact points [8,9]. Dental students and dentists face the problem of improper proximal contact points (PCPs) and proximal overhangs while restoring class II cavities in posterior teeth.

This study aims to compare the operator’s ease and satisfaction of using a CMB system and SMB system on the PCPs and contours when restoring class II cavities in posterior teeth.

## Materials and methods

This cross-sectional study was performed at the clinics in the Department of Conservative Dental Sciences, College of Dentistry, Qassim University. A total of 105 students (60 males and 45 females) randomly participated in this study to restore a class II cavity with direct composite resin restoration (3M ESPE, Seefeld, Germany) from December 2019 to February 2021. Ethical approval was obtained from the ethics committee of the College of Dentistry, Qassim University (Code #: EA/m-2019-3014).

Inclusion criteria were as follows: male and female students of fourth and final year (fifth year) who have restored the proximal cavities with both circumferential and sectional matrix band systems, and all patients who reported to operative clinics, who either had mesial or distal cavities on posterior teeth and were in good general health and have a minimum age of 18 years with fully erupted occluding teeth.

Exclusion criteria were as follows: patients with mesio-occluso-distal (MOD) cavity, teeth with a severe periodontal problem, patients with an orthodontic appliance, diastema between posterior teeth, and tooth mobility of more than score 1. Patients with teeth having class II cavities with the missing adjacent teeth and students who did not use both matrix band systems and restored cavities rejected by faculty specialized in the field of restorative dentistry on overall evaluation were also excluded from the study.

Clinical protocol

The researcher explained the criteria to the operator and made sure that the restorations were placed under a rubber dam (Medesy dental dam). Data were collected from operators who have used both matrix band systems for restoring class II cavities. The questionnaire was filled after asking the operators about the ease, comfort, and satisfaction of using both matrix band systems.

For the teeth that were restored using a CMB system, we used Hahnenkratt (Königsbach-Stein, Germany) contoured Tofflemire bands (no. 101, thickness: 0.04 mm) placed around the tooth in a retainer (Tofflemire Retainer Universal 1140, KerrHawe, Bioggio, Switzerland or AutoMatrix, Dentsply, Konstanz, Germany). Separation was achieved by placing a wooden wedge (set no. 823, sorted; Hawe Neos, Bioggio, Switzerland).

For the teeth that were restored using a pre-contoured SMB system, we used standard Palodent Plus matrix bands (thickness: 0.038 mm; Dentsply, Konstanz, Germany). The SMB was placed inter-proximally and secured with a wooden wedge. Then, the separation ring (Palodent Plus Ring, Dentsply, Konstanz, Germany) was placed.

After matrix placement, excavation, cavity preparation, restoration, and removal of matrix band, a paper questionnaire was filled by the researcher himself for every student after explaining all the variables.

Statistical analysis was performed using the statistical software package SPSS version 25 (IBM Corp., Armonk, NY). We also performed chi‐square tests. A P-value < 0.05 was considered significant at a 95% confidence interval to identify the relationship between the matrix band system and other factors like operators' gender, academic year, and which hand they are using. Descriptive statistics were used to calculate the frequencies and percentages. Missing data were taken into account in our analysis, and there were no missing values.

## Results

A power analysis was performed with alpha at 5% and beta at 20%. In this calculation, the standard deviation within the matrix systems was estimated to be 4, and the standard deviation between the two matrix systems was assumed to be 2.1.

A total of 105 students participated in this study and 51 out of 105 (48.6%) students agreed that CMB required more time for placement as the reason for its difficulty, and 30 out of 105 (28.6%) students agreed that it is not very stable during the procedure. Out of 105 (57.1%) students, 60 agreed that the reason for the difficulty of SMB was the lack of training or experience, and 12 out of 105 (11.4%) students agreed that it required more time for placement. Out of 105 (22.9%) students, 24 agreed that it is not very stable during the procedure (Table 1).

**Table 1:** Reasons of difficulty for CMB and SMB. CMB = circumferential matrix band; SMB = sectional matrix band.

Reasons of difficulty for CMB	Frequency	Percentage	Reasons of difficulty for SMB	Frequency	Percentage
Cumbersome instrument	6	5.7%	Cumbersome instrument	3	2.9%
Lack of proper contour	3	2.9%	Did not face any difficulty with sectional	3	2.9%
Lack of training or experience	12	11.4%	Lack of training or experience	60	57.1%
More time required for placement	51	48.6%	More time required for placement	12	11.4%
Not easily manipulated	3	2.9%	Need good adjacent contact	3	2.9%
Not very stable	30	28.6%	Not very stable	24	22.9%
Total	105	100%	Total	105	100%

Out of 105 (34.4%) students, 36 agreed that the CMB is in the normal range of difficulty, and 63 out of 105 (60%) agreed that the SMB is easy to use. A chi‐square test was done to compare the mean difficulty level with CMB and SMB. A highly significant difference was found between difficulty level and the use of CMB (P < 0.05) and SMB (P = 0.000) (Table 2).

**Table 2:** The difficulty level of using circumferential and sectional matrix bands. 1 = too easy; 10 = too difficult. Levels from 1 to 4 are considered normal range of difficulty. Chi‐square test was used at 95% CI, α = 5%. CI = confidence interval/P-value = 0.000 (P < 0.05). CMB = circumferential matrix band; SMB = sectional matrix band.

Difficulty level of using CMB	Frequency	Percentage	Difficulty level of using SMB	Frequency	Percentage	P-value
1	3	2.9%	1	0	0%	
2	6	5.7%	2	24	22.9%	
3	24	22.9%	3	27	25.7%	
4	3	2.9%	4	12	11.4%	
5	24	22.9%	5	9	8.6%	
6	12	11.4%	6	12	11.4%	
7	15	14.3%	7	9	8.6%	
8	12	11.4%	8	9	8.6%	
9	6	5.7%	9	3	2.9%	
Total	105	100%	Total	105	100	0.000

For the operator’s satisfaction, 54.3% of the operators are satisfied with using CMB for most of their cases while 74.3% of the operators are satisfied with SMB for most of their cases. Also, the chi‐square test was used to find the comparison of operator’s satisfaction with the two types of matrix band systems used. It showed that there were no significant differences between operator satisfaction and the use of the CMB system (P > 0.05) and SMB system (P = 0.134) (Table 3).

**Table 3:** Operator’s satisfaction with using CMB and SMB. Chi‐square test was used at 95% CI, α = 5%. CI = confidence interval/P-value = 0.134 (P > 0.05). CMB = circumferential matrix band; SMB = sectional matrix band.

Operator’s satisfaction with using CMB	Frequency	Percentage	Operator’s satisfaction with using SMB	Frequency	Percentage	P-value
No	48	45.7%	No	27	25.7%	
Yes	57	54.3%	Yes	78	74.3%	
Total	105	100%	Total	105	100%	0.134

A logistic regression analysis was done to evaluate the effect of different variables on the operator’s satisfaction. There was a significant relationship between the operator’s satisfaction and the year-wise distribution of students (Table 4).

**Table 4:** Binary regression analysis to determine the effect of different variables on operator’s satisfaction. CI = confidence interval; SE = standard error; DF = degree of freedom; OR = odds ratio; Sig. = statistical significance.

Different variables may affect operator’s satisfaction	SE	DF	95% CI		OR	Sig.
			Lower	Upper		
Gender	0.444	1	0.408	2.324	0.973	0.952
Year	0.54	1	0.054	0.448	0.155	0.001
Hand	0.854	1	0.270	7.697	1.443	0.668

## Discussion

Mastering proximal tooth-colored restoration with resin composite is very critical. The success of posterior composite restorations is attributed to the operator’s skills, the characteristics of the material, as well as the placement techniques used [10,11]. This cross-sectional study aimed to assess the ease of using matrix band systems for the operators in restoring class II cavities using conventional circumferential and sectional band systems.

Most of the students (60%) agreed that using the SMB system was easier than the CMB system but achieving the optimum position of SMB was unclear to the students due to the lack of training or experience. However, most of the students have good knowledge and experience with CMB but they agreed that it takes time to place (49%), and occasionally it is not stable (29%). Most of the operators were more comfortable with using the SMB in comparison to ones who were using CMB because of its deficiency in establishing optimum contact and recreating the shape of the natural tooth. Nevertheless, some operators have issues in the seal of proximal and gingival walls of the preparation during using SMB.

A study by Loomans et al. in 2006 used two experienced dentists who were well-trained in handling CMB while only one operator was well-trained in using SMB and the other operator was relatively inexperienced. The study showed that no statistically significant differences were found for both operators with various systems [12].

Also, a study done by Durr-E-Sadaf et al. in 2018 found that at our institute (College of Dentistry, Qassim University, Saudi Arabia), “circumferential matrix band with Tofflemire retainer is the only practical method which is used in operative dentistry preclinical year simulation laboratory course” [13].

A study by Chuang et al. that evaluated current matrices and separation systems using three-dimensional imaging concluded that both systems showed problems regarding proximal contour and contact tightness [14]. Thin sectional matrices provided for tight interproximal contacts but concave contours, while circumferential bands produced flat contours but decreased the occurrence of marginal overhangs. The same study also revealed that it was the particular matrix system and not the composite resin material that primarily affected the tooth anatomy and thus the success of treatment [14]. These results match our findings where students had difficulty with both systems especially in creating good contacts and making proper contours.

Separate studies have shown that the use of sectional matrices and separation rings for insertion of class II composite resin restorations resulted in stronger contact surfaces compared to the utilization of traditional circumferential (Tofflemire) bands and wood wedges [10,12,14-17].

Owens et al. concluded in their study that novel developments in matrix system technology have emerged, such as improvements in matrix design and interdental separation techniques. These innovations have allowed the dentist to achieve the most advantageous proximal contact surfaces and anatomically correct contours important for optimum form and function of the dentition as well as for stimulation and protection of the periodontal complex [18].

Our study shows that there are significant differences between difficulty level and the use of CMB system and SMB system and there are no significant differences between operator’s satisfaction with the use of CMB and SMB. In addition, students' year was found to have a significant association with the matrix band system; senior students were more confident, comfortable, and had more experience in using both types of matrix band systems (P = 0.001; P < 0.05).

Despite the good results of using both band systems, training at the undergraduate level for the SMB system was lacking; hence, there was reluctance in using sectional matrix on the part of students. The level of expertise in using matrix band systems increases with the consequent senior year. There could be bias in results for students who did not have enough experience in the application of band systems. The inclusion and exclusion criteria of the study could have been more thorough, taking into consideration more applicable variables, for example, size, location, and level of the cavity may affect the outcome of the results.

## Conclusions

The use of CMB and SMB systems in class II cavities resulted in no statistically significant differences between operator’s satisfaction but using the SMB system was considered easier than using the CMB system. The use of different types of matrix band systems including SMB systems is recommended to be intensified during the laboratory preclinical operative dentistry course.
